# Epicardial adipose tissue differentiates in patients with and without coronary microvascular dysfunction

**DOI:** 10.1038/s41366-021-00875-6

**Published:** 2021-06-25

**Authors:** Ihab Mahmoud, Iryna Dykun, Luisa Kärner, Stefanie Hendricks, Matthias Totzeck, Fadi Al-Rashid, Tienush Rassaf, Amir A. Mahabadi

**Affiliations:** West German Heart and Vascular Center Essen, Department of Cardiology and Vascular Medicine, University Clinic Essen, Essen, Germany

**Keywords:** Preclinical research, Cardiovascular diseases, Body mass index, Risk factors

## Abstract

**Background/Objectives:**

Coronary microvascular dysfunction (CMD) is a common disorder, leading to symptoms similar to obstructive coronary artery disease and bears important prognostic implications. Local inflammation is suggested to promote development of CMD. Epicardial adipose tissue (EAT) is a local visceral fat depot surrounding the heart and the coronary arteries, modifying the inflammatory environment of the heart. We compared EAT in patients with and without CMD.

**Methods:**

We retrospectively included consecutive patients undergoing diagnostic coronary angiography as well as transthoracic echocardiography between March and October 2016. EAT thickness was defined as space between the epicardial wall of the myocardium and the visceral layer of the pericardium and EAT index was calculated as EAT thickness/body surface area. Logistic regression analysis was used to determine the association of EAT index with the presence of CMD.

**Results:**

Overall, 399 patients (mean age 60.2 ± 14.0 years, 46% male) were included. EAT thickness was significantly higher in patients with CMD compared to patients without CMD (EAT thickness 4.4 ± 1.8 vs. 4.9 ± 2.4 mm, *p* = 0,048 for patients without and with CMD, respectively). In univariate regression analysis, EAT index was associated with a 30% higher frequency of CMD (odds ratio [95% confidence interval]: 1.30 [1.001–1.69], *p* = 0.049). Effect sizes remained stable upon adjustment for body mass index (BMI, 1.30 [1.003–1.70], *p* = 0.048), but were attenuated when ancillary adjusting for age and gender (1.17 [0.90–1.54, *p* = 0.25). The effect was more pronounced in patients >65 years of age and independent of BMI and sex (1.85 [1.14–3.00], *p* = 0.013).

**Conclusion:**

EAT thickness is independently associated with CMD and can differentiate between patients with and without CMD especially in older age groups. Our results support the hypothesis that modulation of local inflammation by epicardial fat is involved in the development of CMD.

## Introduction

Epicardial adipose tissue (EAT) is a visceral fat depot that shares direct anatomic contact with the myocardium without fascial interruption. Through its shared blood supply with the coronary circulation, the EAT may cause paracrine effects on the neighboring vasculature and myocardium with inflammatory and atherogenic cytokines, such as monocyte chemo attractant (MCP‐1), interleukin‐β, interleukin‐6, tumor necrosis factor‐α, and leptin [1]. Furthermore, there is an association between EAT and the release of free fatty acids, as well as their myocardial consumption [2].

Substantial evidence has demonstrated that EAT thickness and volume are well-established risk factors for coronary artery disease (CAD) [3–5]. Moreover they offer an accurate and effective estimation for the presence and severity of CAD [6, 7].

Dysfunction of the coronary microvasculature has emerged as an additional mechanism of myocardial ischemia that bears important prognostic implications. Inflammation has been shown to have a pathogenic role in endothelial dysfunction and coronary microvascular dysfunction (CMD) [8].

While extensive data exist toward obstructive CAD, distinct projects evaluating the unique properties of EAT concerning its effects on CMD are currently lacking. Noninvasive assessment of CMD is challenging in clinical routine, as chest pain, suggestive of obstructive CAD, is the key symptom. Therefore, a majority of patients with CMD undergo invasive coronary angiography for suspected obstructive CAD [9]. A previous report on a small selective cohort directs toward EAT thickness being associated with presence of CMD [10]. However, data on larger cohorts, investigating whether noninvasive assessment of EAT thickness, as can easily be quantified from transthoracic echocardiography (TTE), may improve prediction of CMD are currently lacking. In the present study, we aimed to identify whether elevated EAT thickness, as assessed by coronary angiography and TTE, differentiates in patients with vs. without CMD.

## Patients and methods

### Study sample

We retrospectively included consecutive patients from the West German Heart and Vascular Center undergoing diagnostic coronary angiography for suspected CAD but without the need for coronary revascularization therapy as well as TTE between March 2016 and October 2016. Patients with atrial fibrillation, severe mitral valve disease, previous coronary artery revascularization therapy, or left ventricular (LV) ejection fraction <50% were not included. A sample size of 318 was expected to provide 80% power to detect a 20% difference in EAT (type I error rate 5%, 2:1 sampling ratio, estimated mean EAT thickness of 4.5 ± 2.7 mm). The local ethics committee (18-8177-BO) approved the study.

### EAT quantification

**Fig. 1:**
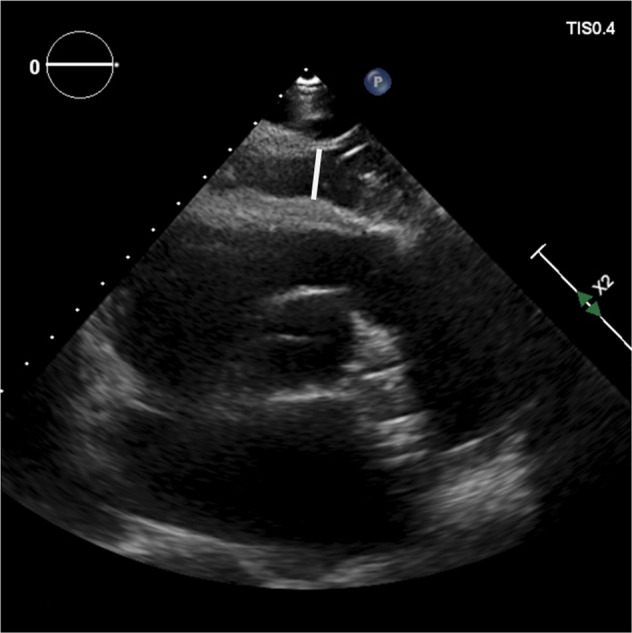
Example image of a EAT measurement in parasternal short-axis view. The EAT thickness is measured as space between the epicardial wall of the myocardium and the visceral layer of the pericardium.

EAT was quantified from TTE. All epicardial fat thickness measurements were performed offline by a single reader, who is highly experienced in both echocardiography and EAT assessment. Epicardial fat was identified as space between the epicardial wall of the myocardium and the visceral layer of the pericardium. Epicardial fat thickness was measured perpendicularly to the free right ventricular wall at end-systole in parasternal long- and short-axis views (Fig. 1). The average value of three measurements was used for EAT thickness. EAT index was defined as EAT thickness normalized to body surface area.

### Risk factor and clinical assessment

Information on risk factors and clinical diagnoses of patients was obtained from all available hospital records. Systolic and diastolic blood pressure was assessed from admission records. Body mass index (BMI) was calculated as weight divided by the square of height, as documented at admission. Total cholesterol, high-density lipoprotein (HDL)-, and low-density lipoprotein (LDL)-cholesterol were measured using standardized enzymatic measures and were recorded from the same hospital stay. Diabetes was defined based on fasting glucose levels, HbA1c levels, or medication. Active smoking and positive family history of premature CAD were assessed as documented by treating physicians. A detailed medication history was obtained including antihypertensive and lipid lowering drugs.

### Left heart catheterization

Left heart catheterization was performed in the supine position using 5F or 6F catheters via the retrograde approach from a femoral or radial artery as previously described [9]. In brief, a pigtail catheter was used for crossing of the aortic valve. LV peak pressure and LV end-diastolic pressures were measured at rest in a steady state. Patients with significant coronary artery stenosis were excluded from the analysis, defined as having lesions ≥70% by diameter in major epicadial arteries (≥50% for left main). For the blood flow assessment, the Thrombolysis in Myocardial Infarction (TIMI) frame out was used [11]. Reading of invasive cardiac hemodynamics and coronary anatomy was performed by experienced invasive cardiologists. CMD was defined using pre-defined combined criteria of LV end-diastolic pressure ≥15 mmHg, or the presence of hypertensive heart disease, or slow flow (TIMI flow ≤II) [9, 11, 12].

### Statistical analysis

Continuous variables are reported as mean ± standard deviation (SD). Discrete variables are given in frequency and percentiles. Baseline characteristics are depicted for the overall cohort and stratified by EAT thickness above vs. below median. Continuous variables were compared using two-sided *T*-test or Mann–Whitney *U* test (for non-normal distributed variables) and discrete variables using Fischer’s exact test. EAT thickness was not normally distributed. Therefore, logistic transformation was performed. Logistic regression analysis was used to determine the association of log-transformed EAT index, with the presence of CMD using the following adjustment sets: model 1: unadjusted; model 2: adjusted for BMI, age, and gender; model 3: ancillary adjusted for systolic blood pressure, HDL- and LDL-cholesterol, diabetes, smoking, and positive family history. Odds ratios and 95% confidence intervals were calculated per each SD of EAT index. In secondary analysis, we stratified by BMI above vs. below median. A *p* value of <0.05 indicated statistical significance. All analyses were performed using SAS software (Version 9.4, SAS Institute Inc.).

## Results

Overall, 399 patients (mean age 60.2 ± 14.0 years, 46% male) were included in our analysis with detailed patient characteristics depicted in Table 1. CMD was present in the majority of patients (*n* = 308, 76.8%).

**Table 1:** Baseline clinical characteristics of the study population stratified by EAT thickness.

	Patients	EAT < median	EAT ≥ median	*p* value
	*N* = 399	*N* = 203	*N* = 196	
Age (years)	60 ± 14	58 ± 14	62 ± 13	0.001
Sex (man)	182 (46%)	91 (45%)	91 (46%)	0.92
BMI (kg/m^2^)	28 ± 6	27 ± 5	28 ± 6	0.15
Systolic RR (mmHg)	135 ± 26	135 ± 26	136 ± 25	0.57
Diastolic RR (mmHg)	69 ± 13	68 ± 13	69 ± 13	0.67
LDL (mg/dl)	126 ± 40	126 ± 42	126 ± 37	0.95
HDL (mg/dl)	55 ± 17	54 ± 15	55 ± 19	0.67
Triglyceride (mg/dl)	145 ± 90	135 ± 77	156 ± 100	0.05
Antihypertensive drugs, *n* (%)	235 (60)	116 (57)	119 (60)	0.48
Lipid lowering drugs, *n* (%)	145 (38)	65 (33)	80 (42)	0.07
Active smoking, *n* (%)	62 (16)	32 (16)	30 (16)	0.89
Diabetes, *n* (%)	57 (19)	27 (14)	30 (16)	0.67
Family history, *n* (%)	68 (18)	33 (17)	35 (18)	0.79
EAT thickness (mm)	4.8 ± 2.2	3.1 ± 0.7	6.5 ± 2	<0.0001

*BMI* body mass index, *EAT* epicardial adipose tissue, *HDL* high-density lipoprotein, *LDL* low-density lipoprotein.

**Fig. 2:**
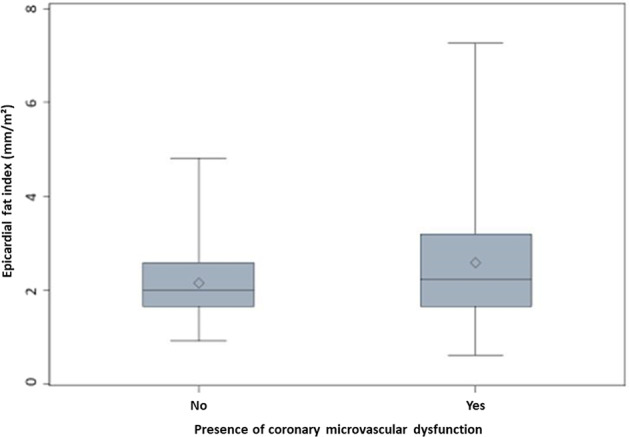
Comparative ability of EAT index to predict CMD. The boxplots show the values of EAT index as divided by the development of CMD.

**Fig. 3:**
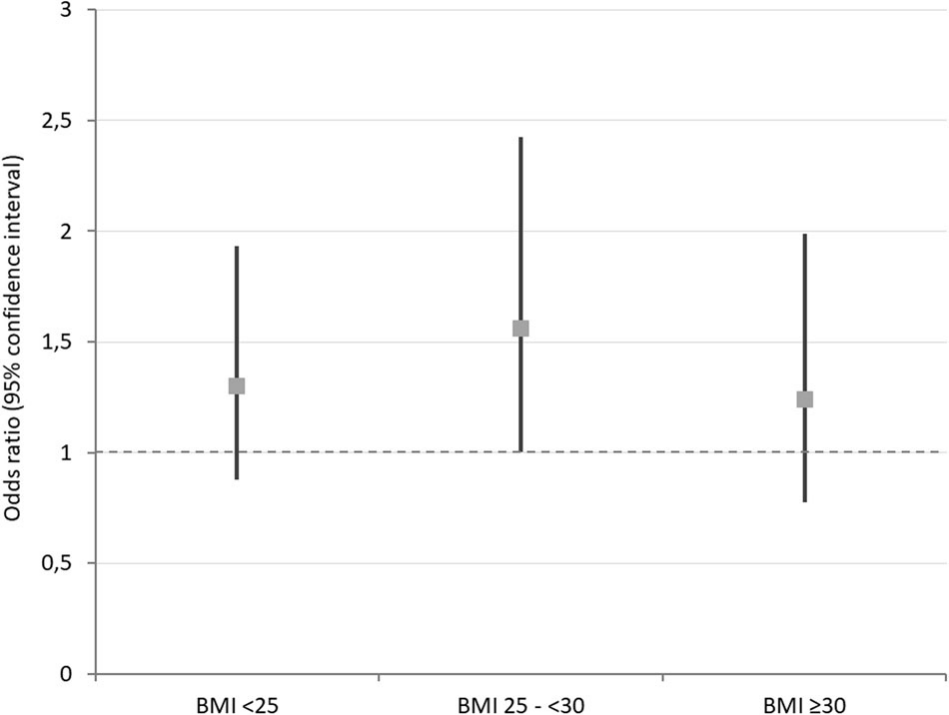
Association of EAT index with the presence of CMD in BMI-stratified analysis. Association of EAT index with presence of CMD was comparable in different BMI subgroups (BMI < 25 kg/m^2^, BMI 25–<30 kg/m^2^, and BMI ≥ 30 kg/m^2^).

EAT thickness was higher in patients with CMD compared to patients without CMD (EAT thickness 4.4 ± 1.8 vs. 4.9 ± 2.4 mm, *p* = 0,048 for patients without and with CMD, respectively). Likewise, EAT index significantly differentiated patients with vs. without CMD (2.1 ± 0.8 mm/m^2^ vs. 2.6 ± 1.3 mm/m^2^, *p* = 0.0003) as depicted in Fig. 2. In a univariate regression analysis, EAT index was associated with a 35% higher frequency of CMD (odds ratio [95% confidence interval]: 1.35 [1.06–1.72], *p* = 0.016 Table 2). Effect sizes remained stable after adjustment for age, gender, and BMI odds ratio [95% confidence interval]: 1.34 [1.04–1.71], *p* = 0.024. Upon adjustment for traditional risk factors, the association of EAT index with CMD was attenuated (Table 2). EAT index as stratified with BMI showed an overlap between different groups with no significant difference (Fig. 3). Stratifying by median BMI, effect sizes were slightly stronger for patients with BMI < median (1.36 [0.95–1.95] vs. 1.24 [0.84–1.82], for BMI < vs. ≥ median, respectively).

**Table 2:** Association of epicardial adipose tissue (EAT) index with CMD in unadjusted and adjusted logistic regression analysis.

Model	OR (95% CI)	*p* value
EAT index	1.35 (1.06–1.72)	0.016
+BMI + Age + Sex	1.34 (1.04–1.71)	0.024
+MV^a^	1.47 (1.03–2.11)	0.035

^a^Multivariable (MV) adjustment includes age, gender, body mass index, systolic blood pressure, high-density and low-density lipoprotein cholesterol, diabetes mellitus, and positive family history of premature cardiovascular disease.

Furthermore, we stratified by age group (<55, 55–<65, and ≥65 years) to assess a potential time-dependent influence of EAT on CMD. We found that EAT was not relevantly associated with CMD in patients aged <55 years, while a strong link was found for patients ≥65 years year so age (Table 3). This association remained stable after adjustment for gender and BMI.

**Table 3:** Association of epicardial adipose tissue (EAT) index with age in unadjusted and adjusted logistic regression analysis.

	Age <55 years		Age 55–65 years		Age >65 years	
	OR (95% CI)	*p* value	OR (95% CI)	*p* value	OR (95% CI)	*p* value
EAT index	0.92	0.68	1.40	0.17	1.76	0.016
	(0.60–1.39)		(0.87–2.26)		(1.11–2.81)	
EAT index + BMI + Sex	0.97	0.89	1.21	0.47	1.85	0.013
	(0.63–1.49)		(0.73–1.99)		(1.14–3.00)	

Odds ratios and 95% confidence intervals calculated per each age group.

*BMI* body mass index, *EAT* epicardial adipose tissue.

## Discussion

In the present study, we investigated the association between EAT thickness and index with the presence of CMD. We found that EAT index was significantly associated with CMD, especially in patients >65 years of age. The link of EAT with impaired microvascular coronary function was independent of general measures of adiposity. Our results support the hypothesis of a local inflammatory impact of epicardial fat that supports the development of CMD over time.

EAT is endocrinally active, secreting several pro- and anti-inflammatory mediators [1]. There is growing evidence suggesting that EAT is related to the extent of coronary plaque burden, and is significantly associated with cardiovascular events and coronary atherosclerosis [6, 7]. Moreover several studies showed that EAT was significantly associated with atrial fibrillation and sever aortic valve stenosis independent of age, gender, and conventional risk factors [13–15]. Persisting inflammation may lead to collagen deposition and subsequent impaired LV relaxation and further effects on diastolic and systolic function. Furthermore, there is an association between EAT and release of free fatty acids, as well as their myocardial consumption [2]. Inflammation, such as in patients with systemic lupus erythematosus and rheumatoid arthritis, has been also associated with CMD [16]. Therefore, EAT and inflammation play an important role in cardiovascular physiology and in the pathogenesis of these diseases. Thus, our study supports the hypothesis that EAT influence the development of CMD via an inflammatory pathway.

Until now, the causes for CMD are not fully explained and most likely multifactorial. More and more evidence of an important role of inflammation in the development of CMD was recently introduced in the literature. The impairment of endothelium-dependent vasodilation and the release of endogenous vasoconstrictor substances such as endothelin-1 have been identified as pathogenic mechanisms in this setting [17]. Moreover, a study in patients with chronic inflammatory conditions with no angiographic CAD or conventional risk factors described the link of inflammation with vascular dysfunction, reduced myocardial blood flow and myocardial ischemia, leading to angina pectoris and CMD [8].

The assessment of coronary flow reserve, the gold standard for assessing CMD, is not only complex but also has several limitations, especially in older patients, and must be regarded with caution [18, 19]. Coronary microcirculation is the major determinant of vascular resistance. Its dysfunction may compromise myocardial perfusion. No technique can visualize the coronary microcirculation in vivo in humans; microvascular function is, therefore, assessed indirectly, through measurements of coronary or myocardial blood flow and coronary flow reserve [20]. Over the last two decades, noninvasive techniques for assessing CMD have evolved. One of these techniques is measuring EAT thickness using TTE, which is reliable, simple, and feasible. Moreover, it showed a very good correlation with EAT measurements with magnetic resonance imaging [21].

Initial data from small and selective cohorts with diverse diagnostic testing suggest that EAT may be increased in patients with CMD [22, 23]. These results are supported by our analysis. While assessment EAT alone may not qualify as reliable diagnostic test, we observed a strong association of EAT with the presence of CMD especially in older patients, a group in whom CMD is frequent and diagnostic according to standardized methods is challenging. Our results therefore suggest that measurement of EAT thickness from TTE may be an easy and broadly available diagnostic opportunity to detect patients at increased risk for CMD.

### Limitations

This study has several limitations that need to be considered. The retrospective nature of our study cohort limits the establishment of causality. The used method is highly dependent on acoustic windows and operator experience. Moreover, the sole quantification of EAT around the right ventricular free wall can be unreliable, as the distribution of adipose tissue around the heart may not be uniform. This problem was avoided by measuring the EAT in two planes. In addition, we did not assess coronary flow reserve as gold standard for the evaluation of CMD, as these are not routinely available in clinical practice. Instead, we used increased LV end-diastolic pressure, coronary slow flow, and hypertensive heart disease as surrogate markers, which are available on every routine coronary angiography. However, given the overlap of long-stenting hypertension, hypertensive heart disease, and CMD, we cannot rule out that the association of EAT with CAD was driven by the presence of these other entities. Finally, echocardiography examination time und coronary angiography were not standardized.

## Conclusion

EAT thickness is independently associated with CMD and can differentiate between patients with and without CMD especially in older age groups. Our results support the hypothesis that modulation of local inflammation by epicardial fat is involved in the development of CMD with early interventions to reduce EAT may protect against the development of CMD. Further studies on larger cohorts are needed to confirm our results.
